# A Photocaged *N*‐Phosphonopiperidinone as a Selective Photo‐Cleavable DPP8/9 Inhibitor

**DOI:** 10.1002/cbic.202500558

**Published:** 2025-09-12

**Authors:** Leonard Sewald, Niko Molke, Werner W. A. Tabak, Anette Haak, Maja Najdzion, Ruth Geiss‐Friedlander, Doris Hellerschmied, Robert Huber, Markus Kaiser

**Affiliations:** ^1^ Chemical Biology Center of Medical Biotechnology Faculty of Biology University of Duisburg‐Essen Universitätsstr. 2 45141 Essen Germany; ^2^ Faculty of Medicine Institute of Molecular Medicine and Cell Research University of Freiburg Stefan‐Meier‐Str. 17 79104 Freiburg Germany; ^3^ Mechanistic Cell Biology Center of Medical Biotechnology Faculty of Biology University of Duisburg‐Essen Universitätsstr. 2 45141 Essen Germany; ^4^ Center of Medical Biotechnology Faculty of Biology University of Duisburg‐Essen Universitätsstr. 2 45141 Essen Germany; ^5^ Emeritus Group Structure Research Max‐Planck‐Institute of Biochemistry Am Klopferspitz 18 82152 Martinsried Germany; ^6^ TUM Senior Excellence Faculty Technical University of Munich Arcisstr. 21 80333 Munich Germany

**Keywords:** activity‐based protein profiling, inhibitors, photocage, proteases, proteomics

## Abstract

The strategic introduction of photocages into chemical probes represents a powerful approach to generate spatiotemporally controlled tools with promising applications in chemical biology and drug discovery. This approach is particularly useful for inhibitors of proteins with cell‐type‐dependent functions, as they enable, via light‐triggered selection, the study of their function in selected cells within multicellular organisms. The intracellular dipeptidyl peptidases 8 and 9 (DPP8/9) are serine hydrolases that act in a cell type‐dependent fashion, in diverse biological processes such as inflammation and tumorigenesis. So far, no photocaged inhibitors for DPP8/9 are available, thus hampering their systematic investigations in biomedical research model systems such as mice. Herein, the development of a green light‐cleavable, BODIPY‐photocaged *N*‐phosphono‐piperidone‐based DPP8/9 inhibitor is presented. This covalent‐acting inhibitor is characterized by its photolysis properties, including a demonstration of its low phototoxicity, as well as potency and selectivity, in biochemical, biological, and, as an extension to these traditional validation approaches, chemical proteomics assays. These studies not only reveal the suitability of the developed photocages for cellular applications, as a prerequisite for their application in multicellular organisms, but also highlight the benefit of chemical proteomics workflows such as activity‐based protein profiling for characterizing proteome‐wide potencies and selectivities of photocaged compounds.

## Graphical abstract

Photocaged inhibitors are versatile biochemical tools for spatiotemporal control of inhibitor activity, facilitating the elucidation of protein function in a cell type‐dependent fashion. Herein, a green light‐cleavable, BODIPY‐photocaged dipeptidyl peptidase 8 and 9 inhibitor with low phototoxicity, pronounced potency, and selectivity is described, thereby demonstrating the benefits of chemical proteomics for inhibitor characterization.

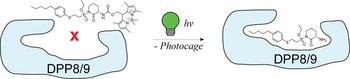

## 1 Introduction

The strategic implementation of photocages, sometimes also referred to as photoremovable protecting groups (PPGs), into chemical tools has emerged as an important strategy to endow chemical inhibitors with further spatiotemporal control. Such probes are then transformed into their active form by irradiation with light, resulting in a controlled release of the PPG.^[^ ^1^, ^2^ ^–^ ^3^ ^]^ Accordingly, PPGs are photosensitive chromophores that are cleaved by light at specific wavelengths. The most often explored PPGs are *o*‐nitrobenzyl‐based systems, which are activated by UV light irradiation, thereby, however, limiting their applicability in many biological systems due to UV phototoxicity and low tissue penetration.^[^ ^4^, ^5^ ^–^ ^6^ ^]^

In recent years, substantial efforts have resulted in PPGs that no longer require UV light for cleavage but can be released by irradiation with light of longer wavelengths, e.g., in the visible (VIS) range, thereby overcoming previous limitations.^[^ ^2^ ^,^ ^3^ ^,^ ^7^ ^,^ ^8^ ^]^ Indeed, the recent advances in the development of PPGs have raised hopes that even clinically useful photocaged therapeutics may become possible in the future.^[^ ^9^ ^]^ Among the different VIS PPGs developed so far, *meso*‐methyl BODIPY photocages have thereby become one of the most often used modalities. These photocages combine tunable absorption properties from 500 to 700 nm, important for low phototoxicity and deep tissue penetration, with a promising cell membrane permeability and the possibility to further modify them with subcellular compartment targeting moieties.^[^ ^10^ ^]^ The application of this photocleavable modality has, for example, been used in the development of the photocleavable cathepsin B inhibitor CA‐074 that showed promising biochemical and biological properties with high phototherapeutic indexes.^[^ ^11^ ^]^ Despite these advances, a more systematic use of photocaged inhibitors in biomedical research is, however, limited by the restricted availability of characterized PPG inhibitors and of streamlined approaches to characterize these photocaged variants. Indeed, for most relevant target proteins, no photocaged inhibitors are available at all, and for the few established photocaged inhibitors, many of their inhibitory properties, such as proteome‐wide selectivities, are missing.^[^ ^12^ ^]^

A promising application for VIS‐cleavable photocages is the development of PPG‐modified dipeptidyl peptidase (DPP) 8 and 9 (DPP8/9) inhibitors. These intracellular, highly homologous serine proteases of the dipeptidyl peptidase 4 activity/structure homologs family cleave post‐proline bonds at the penultimate position of the N‐terminus of a substrate.^[^ ^13^, ^14^ ^–^ ^15^ ^]^ They are involved in diverse cell biological processes such as pyroptosis or ubiquitin‐mediated proteolysis and have been proposed as potential target proteins for antiinflammatory and anticancer therapy.^[^ ^15^, ^16^, ^17^, ^18^, ^19^ ^–^ ^20^ ^]^ Originally, both proteins were thought to display mainly overlapping physiological functions, while recent studies indicate that both enzymes also overtake individual functions. For example, DPP9 plays a key role in inflammatory cell death via pyroptosis induction, while DPP8 appears to be less relevant.^[^ 21, 22, 23, 24, 25, 26 ^]^ Furthermore, DPP9 is essential for neonatal survival,^[^ ^27^ ^]^ memory retrieval and long‐term potentiation in mice,^[^ ^28^ ^]^ regulates B cell signaling^[^ ^29^ ^]^ and repair of DNA double‐strand breaks.^[^ ^30^ ^]^ Due to the distinct functions that DPP8 and DPP9 overtake in diverse cell types, e.g., their specific function in immune cells or in cancer cells, an approach to induce their cell‐type‐specific inhibition in a more complex model organism would be highly desirable. We therefore report here the development of the first VIS cleavable photocaged DPP8/9 inhibitors, including a characterization of their inhibitory traits such as photocleavage parameters, potency, and selectivity in cells, as a prerequisite for a subsequent evaluation of these probes in multicellular systems such as tissues or even whole model organisms. To this end, we combined photocleavage experiments with biochemical, biological, and cellular proteomics assays.

**Scheme 1:**
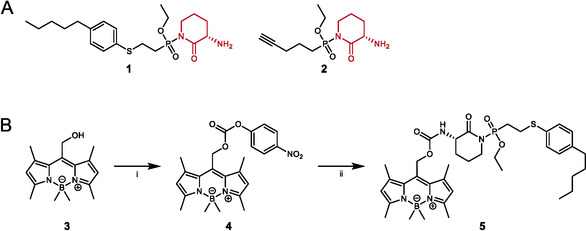
Chemical structure and synthesis of compounds used in this study. A) Chemical structure of *N*‐phosphono‐(*S*)‐3‐aminopiperidine‐2‐ones analogs used in this study. The (*S*)‐3‐aminopiperidine‐2‐one leaving group is shown in red. B) Chemical synthesis of photocaged inhibitor **5** via conversion of a boron‐dimethyl BODIPY photocage (**3**) to a *para*‐nitrophenol carbonate derivative (**4**) and subsequent coupling to **1**. Reagents and conditions: i) DCM, DIEA, 4‐nitrophenylchloro formiate, 16 h, rt; ii) **1**, DMSO, DIEA, 4 h, rt.

To obtain such photocaged DPP8/9 inhibitors, we make use of our recent findings on the development of covalent DPP8/9 inhibitors.^[^ ^31^ ^]^ We reported the identification of *N*‐phosphono‐(*S*)‐3‐aminopiperidine‐2‐ones, rationally designed derivatives of the natural product sulphostin, as potent and selective covalent DPP8/9 inhibitors. Of them, a 2‐(4‐pentylphenylthio)ethyl‐modified analog (**1**, **Scheme** **1**A) was the most active and selective inhibitor, inhibiting DPP8 slightly more strongly than DPP9. Its proteome‐wide selectivity was thereby confirmed with the help of an alkyne‐tagged derivative (**2**) that was used in a competitive activity‐based protein profiling (ABPP) approach.^[^ ^31^ ^]^ Here, we developed this inhibitor structure into a PPG derivative by attaching the photocage, a boron‐dimethyl BODIPY moiety, to the (*S*)‐3‐aminopiperidin‐2‐one leaving group (Scheme 1B). By performing biochemical, biological, and competitive ABPP experiments, we then show that this design leads to a promising photocaged inhibitor that exhibits potent target binding only after activation by green light irradiation.

## 2 Results

### 2.1 Chemical Synthesis of a Photo‐Cleavable Covalent DPP8/9 Inhibitor

To generate a photo‐cleavable covalent DPP8/9 inhibitor, we made use of our previous identification of *N*‐phosphono‐piperidones as potent and selective DPP8/9 inhibitors.^[^ ^31^ ^]^ These inhibitors act by an unusual mode of action, inducing a covalent modification of the active site serine by the phosphonate moiety via a concomitant release of the piperidone residue and thus of the moiety of the inhibitor that triggers target selectivity (Scheme 1A). After synthesis of a ‘Winter Green’ boron‐dimethyl BODIPY photocage building block **3**, by following essentially published synthesis routes (Scheme S1, Supporting Information),^[^ ^32^ ^,^ ^33^ ^]^ **3** was converted into a *para*‐nitrophenol carbonate derivative **4** and then coupled to **1**, a potent and selective DPP8/9 inhibitor. Coupling was thereby achieved by a simple mixing of **1** and **4** in mildly basic conditions, thereby delivering the desired ‘Winter Green’ BODIPY photocage (**5**, Scheme 1B) in good overall yields.

**Figure 1:**
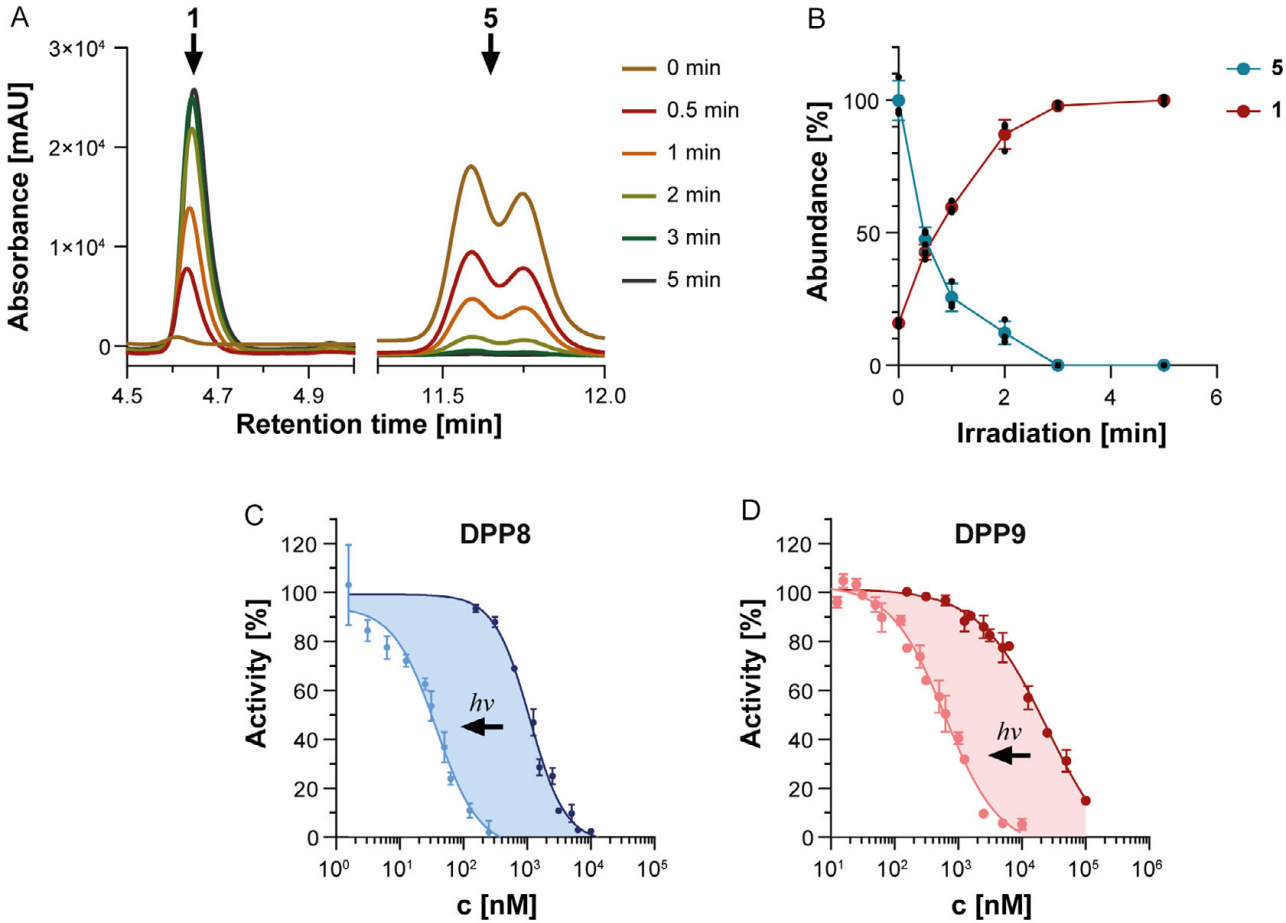
DPP inhibition by photo‐cleavable inhibitors. A) LC‐MS analysis of the photocleavage reaction of **5** after irradiation with green light. 50 µM of compound **5** was irradiated with a green LED lamp (*λ *= 520‐530 nm, 30 W) for up to 5 min, and the resulting reaction mixture was analyzed via LC‐MS. Depicted is the corresponding UV (*λ *= 254 nm) trace of these runs at selected retention times, corresponding to the elution of the photocleaved product (corresponding to **1**) at t_R_ of 4.65 min and of the photocaged starting material **5** at *t*_R_ of 11.5–11.9 min. B) Resulting quantification of the photolysis of **5** based on peak areas depicted in A) (*n* = 3 technical replicates). C,D) DPP8 and DPP9 inhibition curves of **5** after (light blue or red) or without (dark blue or red) green light irradiation. Enzyme activity was determined by measuring the release of AMC from the substrate GP‐AMC. All measurements were performed in triplicate (*n* = 3 technical replicates), mean values are shown, and the error bars indicate the standard error of the mean (SEM). Data was normalized to the DMSO control.

With **5** in our hands, we then started our probe characterization by determining the velocity of the photorelease reaction, i.e., the light‐triggered cleavage of the photocage, thereby delivering again the “active” inhibitor **1**. To this end, a 50 µM solution of **5** in 10% acetonitrile in MS‐grade water was irradiated with a green LED lamp (*λ *= 520‐530 nm, 30 W) for the indicated time periods, and the release of the active inhibitor **1** was quantified via liquid chromatography‐mass spectrometry (LC–MS) analysis (**Figure** **1**A). These measurements revealed a rapid photorelease reaction in which, already after 2 min, 95% of the photocage was cleaved off (Figure 1B). After 3 min, cleavage was quantitative (>99%). Importantly, the only products observed were the desired ‘free’ inhibitor **1** and the cleaved photogroup, indicating a clean activation to **1**. Longer irradiation times did not lead to further side products, indicating the photostability of inhibitor **1** in the employed photorelease reaction conditions. Altogether, these experiments show that **5** can be rapidly and quantitatively converted into the active inhibitor **1**, without the need for any specific precautions for preventing photodamage to **1**.

### 2.2 DPP Inhibition by 5 is Dependent on Activation by Irradiation

We then continued to investigate the biochemical properties of the photocaged compound **5** versus the photoreleased product resembling inhibitor **1**. To this end, we performed biochemical enzyme inhibition assays with DPP8 and DPP9 before and after irradiation of **5**. Resulting DPP8/9 inhibition was quantified by following the DPP8‐ or DPP9‐mediated proteolysis of the fluorophore 7‐amino‐4‐methylcoumarin (AMC) from the model substrate Gly‐Pro‐AMC (Figure 1C,D and **Table** **1**). To induce photocleavage of **5**, reaction mixtures were irradiated for 5 min with the same green LED lamp as before, followed by a pre‐incubation of 45 min with the photoreleased inhibitor prior to substrate addition. We observed that irradiation of **5** with green light led to significantly more potent DPP8 and DPP9 inhibition by **5**, whereas DPP4 was not inhibited under both irradiated and non‐irradiated conditions. More specifically, without irradiation, an apparent IC_50_ of 1,095 ± 66 nM for DPP8 and of 24.7 ± 6.8 µM for DPP9 was determined. However, after green light‐mediated photorelease, an apparent IC_50_ of 37 ± 3 nM for DPP8 and of 621 ± 54 nM for DPP9 was observed, corresponding to an about 30‐ and 40‐fold increase in potency, respectively, which aligns well with the performance of other photocaged protease inhibitors and is in agreement with literature values for the parent compound.^[^ ^11^ ^,^ ^31^ ^,^ ^34^ ^,^ ^35^ ^]^ Importantly, these assays also revealed the existence of suitable concentration ranges that lead to full inhibition after photorelease and almost no inhibition without prior irradiation. Finally, these results not only demonstrate the successful introduction of a photocage in the potent DPP inhibitor structure but also show that irradiation with green light has no effect on protein activity.

**Table 1:** Apparent IC_50_ [nM] values for inhibition of DPP4/8/9 by photocaged inhibitor 5 after or without green light irradiation.

		Apparent IC_50_ [nM]	Dark/light ratio
DPP4	Dark	>100,000	–
	Light	>100,000	
DPP8	Dark	1,095 ± 66	30
	Light	37 ± 3	
DPP9	Dark	24,684 ± 6,767	40
	Light	621 ± 54	

As previously shown by us, **1** inhibits DPP8 and DPP9 via a covalent modification of the active site serine.^[^ ^31^ ^]^ Therefore, we also measured the time dependence of this covalent modification of the target enzyme by recording progress curves for the inhibition of DPP8 and 9 and using them to calculate the corresponding binding affinity (K_I_), maximum enzyme inhibition rate (k_inact_) as well as the efficiency of covalent bond formation (k_inact_/K_I_) values for **5** after and without green light irradiation ( Figure S1 and Table S1, Supporting Information). These measurements revealed a hyperbolic k_obs_ versus inhibitor concentration curve characteristic of covalent inhibitors. In agreement with the previous inhibition experiments, a significantly stronger inhibition of the enzymes was found under “light” conditions. In detail, DPP8 was inhibited with a k_inact_/K_I_ of 8,540 M^−1 ^s^−1^ and DPP9 with a k_inact_/K_I_ of 737 M^−1 ^s^−1^. By contrast, under “dark” conditions, DPP8 was only inhibited with a k_inact_/K_I_ of 223 M^−1 ^s^−1^ and DPP9 with a k_inact_/K_I_ of 14 M^−1 ^s^−1^, correlating to about 38‐ and 51‐fold less potent inhibition, respectively.

### 2.3 Competitive ABPP with Photo‐Cleavable DPP Inhibitors

**Figure 2:**
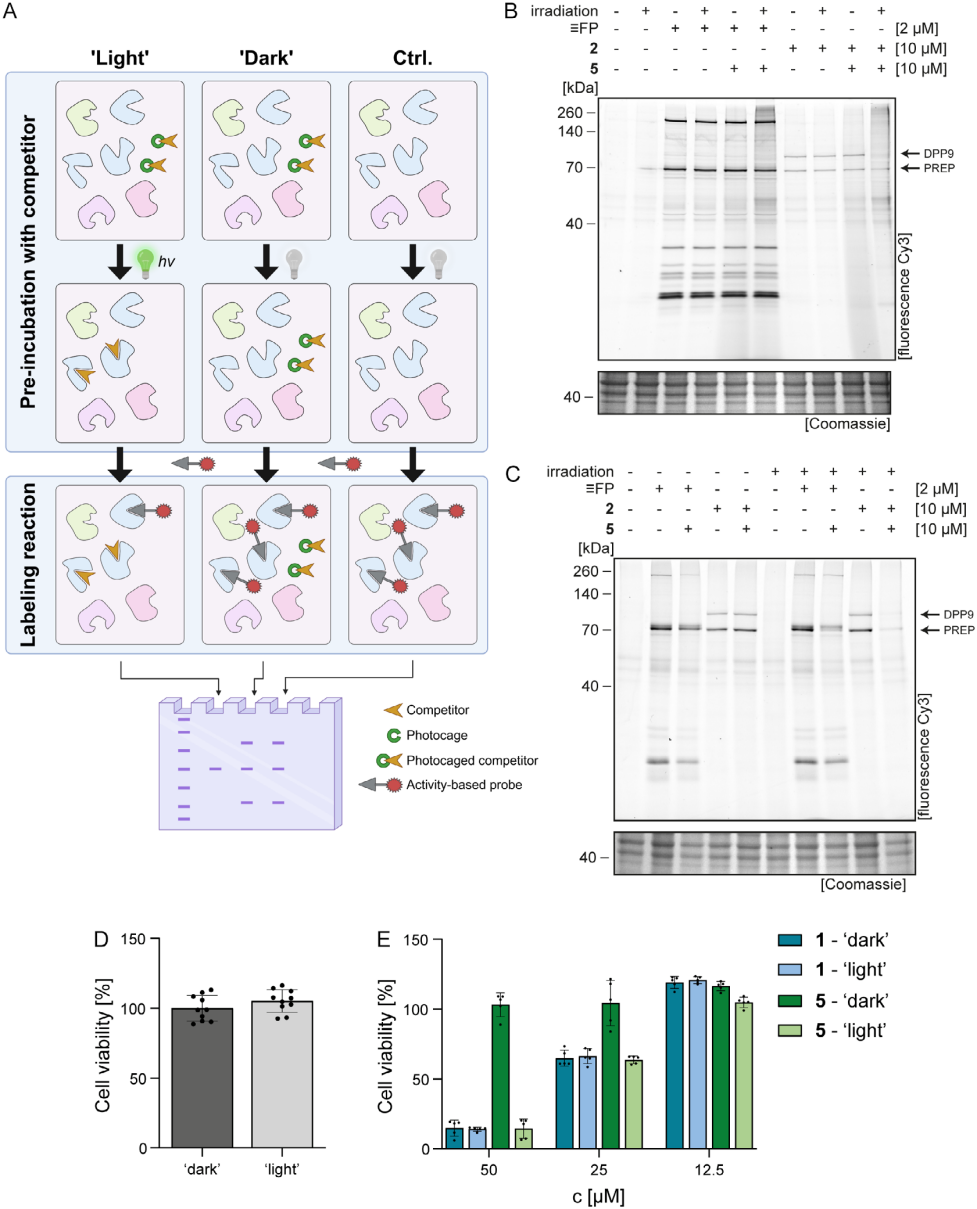
Competitive ABPP with photo‐cleavable inhibitor **5** in HEK293 cells. A) Schematic overview of a competitive ABPP experiment. A proteome is pre‐incubated with a competitor, followed by a labeling reaction with an activity‐based probe (ABP). In the case of a photocaged inhibitor, the active compound can be released via irradiation. Target proteins of the competitor cannot be bound by the ABP, resulting in an absence of the respective protein band in‐gel analysis. Created in BioRender. Najdzion, M. (2025) https://BioRender.com/84zn8cb B) Gel‐based competitive ABPP experiment in HEK293 cell lysates with the broadband serine hydrolase ABP **≡FP** or the DPP8/9‐directed ABP **2** and the photocaged inhibitor **5**, at the indicated concentrations, after or without prior irradiation with green light for 5 min. The corresponding pre‐incubation time for **5** was 30 min. C) Corresponding in situ competitive ABPP experiment in HEK293 cell culture, again with 10 µM **2** for 1 h. Labeling was competed by pre‐incubation with 20 µM **5** for 1 h, followed by an activation of the photocaged inhibitor by irradiation for 5 min with a green LED lamp, if applicable. D) Green light treatment has no cytotoxic effect. HEK293 cells were irradiated with a green LED lamp in the presence of 0.5% DMSO for 5 min (‘light’), and cell viability was measured after 24 h. The graph shows the mean ± SEM of all individual measurements (*n* = 10 biological replicates). Data was normalized to the non‐irradiated control (‘dark’). E) Green light treatment leads to the release of the active DPP8/9 inhibitor. HEK293 cells were incubated with compound **1** or **5** for 24 h. For both compounds, cell viability was measured under ‘dark’ (not irradiated) and ‘light’ (irradiated for 5 min with a green LED lamp after the addition of the inhibitor) conditions. The graph shows the mean ± SEM of all individual measurements (*n* = 5 biological replicates). Data was normalized to the non‐irradiated DMSO control.

After showing that the photocaged compound **5** displayed only weak inhibitory properties against DPP8/9 at a low micromolar concentration without irradiation, we next tested the applicability of the photocleavable inhibitor within a proteome in the course of a competitive ABPP experiment (**Figure** **2**A). Therefore, HEK293 cell lysates were pre‐incubated with 10 µM **5** or DMSO vehicle control for 30 min, followed by a labeling reaction with 2 µM of the serine hydrolase‐specific ABP fluorophosphonate‐alkyne (**≡FP**) or 10 µM **2** (Figure 2B). As expected, **≡FP** application with and without prior irradiation led to the same band pattern, showing again that photocleavage with green light does not lead to photodamage to proteins. We then performed a competitive ABPP approach in which we observed that incubation with **5** without any irradiation did not result in any competition, whereas pre‐incubation with **5**, followed by green light irradiation, resulted in a labeling competition of one specific band with a molecular weight (MW) of about 100 kDa. Of note, this band has been previously assigned as DPP9 (DPP8 would migrate in the same range, but is only expressed at low levels in the used cell line and therefore cannot be visualized by this approach).^[^ ^36^ ^,^ ^37^ ^]^ A corresponding ABPP experiment with **2** revealed two bands, one again at ca. 100 kDa and therefore corresponding to DPP9 as well as one band at 70 kDa, which most probably corresponds to prolyl endopeptidase (PREP), which is an off‐target protein of the *N*‐phosphono‐piperidone probe **2**, but to a much lesser extent of **1**, as previously shown by us.^[^ ^31^ ^]^ Again, this labeling was observed regardless of prior irradiation. Subsequently, the competitive labeling experiment was conducted by pre‐incubation with compound **5**, with or without subsequent irradiation. Notably, efficient DPP9 labeling competition was detectable only following irradiation, evidenced by the loss of the 100 kDa band. We also observed a slight reduction of the supposed PREP band, again in agreement with the weak off‐target activity of **1** for this enzyme. Altogether, these experiments reveal that the photocaged inhibitor indeed shows promising selectivities in cell lysates.

To confirm its suitability also for living cells, we performed the corresponding competitive ABPP experiments with a HEK293 cell culture, consisting of in situ application of **5** and the corresponding labeling probe, irradiation, and thus a photo‐mediated release of the active inhibitor in cells, followed by the standard 2‐step ABPP procedure to subsequently visualize the outcome of the ABPP experiment (Figure 2C). The corresponding in‐gel detection with both ABPs **FP** and **2** resulted in a comparable band pattern as for the labeling experiment with lysates. Again, labeling competition of only two prominent bands at about 70 and 100 kDa was observed after pre‐treatment with **5** and irradiation with green light for 5 min. Whereas the labeling of PREP was partially competed, labeling of DPP9 was completely inhibited by application of the photocleavable inhibitor under “light” conditions. Altogether, these experiments confirm that photorelease and thus activation of **5** can also be performed in live cells. In addition, the high inhibition selectivity ratio between caged and uncaged **5** is also maintained in live cells, thereby opening the possibility to apply **5** to a whole cell culture and then to induce selective inhibition of DPP8/9 in selected cells via green light treatment.

### 2.4 Target Engagement of Photo‐Cleavable DPP8/9 Inhibitors in Cells

In order to validate target engagement in living cells and potential cytotoxic effects of green light treatment, we determined cell viability of HEK293 cells after 24 h inhibitor treatment with **5** with or without 5 min green light irradiation, as the application of DPP8/9 inhibitors at high concentrations and thus elimination of any residual DPP8/9 activity results in cytotoxicity.^[^ ^38^, ^39^ ^–^ ^40^ ^]^ As a control, effects of green light treatment on cell viability were examined in the absence of any inhibitor after 24 h, again with or without 5 min irradiation (Figure 2D). First, these studies revealed no cytotoxicity from the green light treatment. Moreover, consistent with our previous study,^[^ ^31^ ^]^ the parent ‘free’ inhibitor **1** did not significantly affect cell viability in the low micromolar range, while cytotoxic effects were observed after application of 25 and 50 µM of **1**, resulting in approximately 65% or 15% remaining viability, respectively, regardless of irradiation (Figure 2E). By contrast, the photocaged inhibitor exhibited no cytotoxicity under “dark” conditions, even at the highest concentrations used in the assay. However, after irradiation, the viability of cells treated with 25 and 50 µM **5** was significantly reduced, and importantly, to a comparable level as observed for application of **1**, suggesting a successful photolysis and subsequent target engagement. Of note, no additional effect of the photocage on cell viability was observed. In summary, our cell viability studies confirm target engagement in live cells exclusively after photorelease, thereby again confirming the suitability of the photocaged DPP8/9 inhibitor **5** as a cell biological tool compound.

## 3 Discussion

The introduction of PPGs into bioactive small molecules represents a promising approach for obtaining inhibitors with spatial and temporal control of their activity. On a longer view, photocaged inhibitors could even offer new therapeutic possibilities, as targeted partial activation, e.g., exclusively in certain compartments, cells, or tissues, could increase selectivity and reduce undesirable side effects. The use of photocages that can be cleaved with visible light is particularly promising. It is therefore surprising that, despite their advantages, only a limited number of fully characterized photocaged inhibitors have been reported to date. Moreover, among this already small set of compounds, the majority still rely on UV‐cleavable PPGs.

To achieve selective inhibition, i.e., weak inhibition of the target by the photocaged compound but potent inhibition after photorelease, photocages are typically attached to regions of an inhibitor that are critical for interaction with the target protein. In the case of protease inhibitors, this strategy often involves designing a photocaged derivative that, due to steric hindrance, is unable to bind the active site of the protease.^[^ ^12^ ^]^ Upon photocleavage, this steric block is removed, allowing the inhibitor to access the active site and exert its inhibitory effect. This approach has been employed, for example, in the development of photocaged inhibitors targeting caspases,^[^ ^34^ ^]^ cathepsins,^[^ ^11^ ^,^ ^35^ ^]^ and the proteasome.^[^ ^41^ ^]^ We have also adopted this design strategy; however, in our case, the photocage was attached to the inhibitor's leaving group, a moiety important for binding but no longer present in the final inhibitor–protein complex following covalent modification. Our findings demonstrate that such a design can also yield effective photocaged inhibitors.

Furthermore, we show that the application of modern chemical proteomics techniques, such as competitive ABPP, provides valuable insights into the key properties of photocaged inhibitors, including their proteome‐wide potency and selectivity before and after photorelease. Given its efficiency and informative value, we propose that this technology should be more widely used in the characterization of photocaged probes.

Our cell biological studies with the photocaged DPP8/9 inhibitor have also shown that **5** might represent a promising tool for the elucidation of DPP8/9 function in certain cells and tissues through temporal or spatial control of the inhibitor activity. Previously, it was shown that DPP8 and DPP9 may be involved in tumor progression,^[^ ^42^ ^]^ growth and metastasis^[^ ^20^ ^,^ ^43^ ^,^ ^44^ ^]^ leading to poor prognosis for various cancers in combination with high levels of DPP9.^[^ ^15^ ^,^ ^17^ ^,^ ^45^ ^,^ ^46^ ^]^ By contrast, high DPP8/9 expression correlates with a good prognosis in breast cancer patients,^[^ ^30^ ^,^ ^43^ ^,^ ^47^ ^]^ and global DPP9 activity is critical for neonatal survival,^[^ ^27^ ^]^ memory,^[^ ^28^ ^]^ DNA repair,^[^ ^30^ ^]^ and mitochondria homeostasis^[^ ^48^ ^]^ emphasizing the potential of a temporal and spatial DPP8/9 inhibitor for elucidation of enzyme function in certain organs, tissue or cells. We anticipate that the developed photocaged inhibitor can be employed to better study these processes and should, after our promising results, now also be evaluated in multicellular model systems such as mice.

## 4 Experimental Section

4.1

4.1.1

##### Chemical Synthesis

Chemical syntheses of all compounds from this study are reported in the Supplementary Information.

##### Quantification of Photolysis

Compound **5** was dissolved in DMSO. In order to quantify the effect of irradiation time on photocleavage of the BODIPY moiety, 50 µL aliquots of a 50 µM solution of **5** in 10% acetonitrile (ACN; VWR Chemicals, USA) in MS‐grade water (VWR Chemicals, USA) were irradiated for 0–5 min with a green LED lamp (*λ *= 520–530 nm, 30 W; Eurolite, Germany) at room temperature (RT) and gentle shaking (final DMSO concentration: 0.5%).

Quantification of inhibitor release was then determined via LC‐MS on an LTQ XL linear ion trap mass spectrometer (ITMS; Thermo Fisher Scientific, USA) coupled to a DIONEX UltiMate 3000 system (Thermo Fisher Scientific, USA), operated using Xcalibur software 4.4.16.14. The analytical column (NUCLEODUR C18 Pyramid, 5 µm, 250 × 4 mm; Macherey‐Nagel, Germany) was encased by a column oven (25 °C). Analytes were separated using a 19 min gradient of solvent A (0.1% formic acid (FA) in MS‐grade water) and solvent B (0.1% FA in ACN) (start with 10% B for 0.5 min, gradient 10% to 40% B for 0.5 min, gradient 40% to 100% B for 5 min, 100% B for 10 min, gradient 100% to 10% B for 0.4 min, 10% B for 2.6 min) with a flow rate of 1 mL min^−1^ and UV spectra were recorded at 254 nm. The mass spectrometer was operated in the positive ion mode with a scan range of 110–2,000 *m/z* and a normal scan rate. The ion scanning (MS1) was done in an ITMS. Quantification was done using Freestyle v1.6. based on the UV spectra (*λ *= 254 nm) using the Avalon peak detection algorithm. All measurements were performed in triplicate.

##### Biochemical Assays

Enzyme activity of human DPP4, DPP8, and DPP9 (Proteros Biostructures GmbH, Germany) was determined by measuring the initial velocity of AMC release from the fluorogenic substrate GP‐AMC (Sigma Aldrich, USA) using a Tecan Spark 10 M multimode microplate reader (Tecan Group Ltd., Switzerland) in activity buffer (20 mM HEPES/KOH pH 7.3, 110 mM potassium acetate, 2 mM magnesium acetate, 0.5 mM EGTA, 0.02% Tween 20, supplemented with 1 mM DTT) and a final volume of 20 μL for maximal 30 min at 30 °C. Prior to the activity measurement, 10 nM of the corresponding enzyme was incubated for 45 min at 30 °C and gentle shaking with the indicated compounds at concentrations ranging from 0 to 100 μM, followed by irradiation with a green LED lamp (*λ *= 520−530 nm; Eurolite, Germany) for 5 min. The corresponding compounds were dissolved in DMSO and diluted in activity buffer (final DMSO concentration: 1%). To the inhibitor–enzyme mixture, GP‐AMC was then added to a final concentration of 250 μM, and the activity measurement was started. The resulting enzyme activity was calculated from the slope (fluorescence release over time) and plotted against the concentrations of the inhibitor. Apparent IC_50_ values were calculated using Graph‐Pad Prism v10.4.1 using the equation: [Inhibitor] versus response‐Variable slope (four parameters). All measurements were performed in triplicate, and error bars represent standard errors of the fit.

To calculate the reaction rate of covalent bond formation between DPP proteins and the inhibitor, k_inact_, K_I_, and k_inact_/K_I_ were determined as previously described.^[^ ^49^ ^,^ ^50^ ^]^ To this end, a dilution series of **5** was irradiated using a green LED lamp (*λ *= 520−530 nm; Eurolite, Germany) for 5 min and subsequently mixed with 250 µM GP‐AMC. The mixture was then added to 10 nM enzyme, and the resulting activity was measured for a maximum of 60 min at 30 °C. Fluorescence intensities were plotted against time and data were fitted to the following equation: 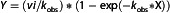 using Graph‐Pad Prism v10.4.1. The obtained pseudo first‐order rate constant was used to determine k_inact_ and K_I_ by fitting the plot of k_obs_ against inhibitor concentration to the following equation: 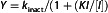. The efficiency of covalent bond formation (k_inact_/K_I_) was calculated by the ratio of k_inact_/K_I_. All measurements were performed in triplicate, and error bars represent standard errors of the fit.

##### Cell Culture

For cell‐based experiments, HEK293 cells (Cytion, Germany, 300192) were cultured in Dulbecco's modified Eagle's medium (DMEM, Gibco, USA) supplemented with 10% fetal bovine serum (FBS, Gibco, USA) and 1% Penicillin/Streptomycin (Gibco, USA) at 37 °C under a humidified 5% carbon dioxide atmosphere. Cells were cultured in 10 cm tissue culture dishes. A sub‐cultivation ratio of 1:4 to 1:5 was used for passaging cells every 2–3 days when cells reached ≈80–90% confluence.

##### In Vitro Competitive ABPP

For in vitro ABPP, cell lysates were generated from HEK293 cells grown on a 10 cm tissue dish. Cells were washed with phosphate‐buffered saline (PBS, Gibco, USA), detached with trypsin (Gibco, USA), and pelleted via centrifugation (5 min, 250 × *g*, RT), followed by three consecutive wash steps with PBS. Cell pellets were re‐suspended in 400 μL phosphate buffer (50 mM HNa_2_PO_4_, pH 8) and lysed by sonication (Bioruptor Plus, Diagenode, Belgium; 1 min pulse and 0.5 min pause in 10 cycles with high power). The cell lysates were cleared via centrifugation (30 min, 20,817 × *g*, 4 °C), and protein concentration was determined with a modified Bradford assay using Roti‐Nanoquant (Carl Roth, Germany). For labeling competition, 50 µg protein was pre‐incubated with 10 µM or DMSO as a vehicle control (30 min, 37 °C, gentle shaking) and, if applicable, irradiated with a green LED lamp for 5 min (after addition of the competitor). Proteins were then labeled with 2 µM ≡**FP** or 10 µM **2** (1 h, 37 °C, gentle shaking) and subjected to a click reaction with 10 μM Cy3‐N_3_ (synthesized in house), 100 μM TBTA (Sigma Aldrich, USA), 2 mM TCEP (Sigma Aldrich, USA), and 1 mM CuSO_4_ (Sigma Aldrich, USA; 1 h, RT, in the dark).

##### In Situ Competitive ABPP

For in situ ABPP experiments, 2 × 10^5^ cells were seeded on a 24‐well plate and incubated for 24 h as described before. Prior to treatment, the cell culture media were replaced by fresh 0.5 mL DMEM. For labeling competition, cells were pre‐incubated with 10 µM **5** or DMSO as a vehicle control (60 min, 37 °C) and, if applicable, irradiated with green light for 5 min (after addition of the competitor). Cells were then treated with 10 µM **2** (60 min, 37 °C) and harvested as described before. In brief, culture supernatant was transferred into a fresh reaction vessel, and cells were washed with 500 µL PBS. Cells were detached by 300 µL trypsin and then re‐suspended in 700 µL DMEM. Cells were pelleted via centrifugation (5 min, RT, 400 × *g*) and washed thrice with 1 mL PBS. The cell pellet was re‐suspended in 50 µL phosphate buffer (50 mM HNa_2_PO_4_, pH 8), and protein concentration determination, as well as subsequent click reaction with a total protein amount of 50 µg was performed as described above.

##### Gel Electrophoresis Analysis

The click reaction was stopped by adding one equivalent 4× gel loading dye (280 mM sodium dodecyl sulfate (SDS), 400 mM Tris base, 40% (w/v) glycerol, 1.4 M *β*‐mercaptoethanol, 0.6 mM bromophenol blue, pH 6.8) and incubating at 95 °C for 5 min prior to separation of proteins by SDS‐polyacrylamide gel electrophoresis (6% stacking and 12% separating gel; 140 V, 55 mA, 1.5 h). Visualization of fluorescently labeled proteins was performed using a Typhoon FLA 9000 laser scanner (GE Healthcare, USA). Subsequently, proteins were stained with colloidal Coomassie (0.757 M ammonium sulfate, 0.1% (w/v) Coomassie G‐250, 2.55% orthophosphoric acid, 20% ethanol) and visualized with an Intas140S gel documentation system (Intas, Germany).

##### Cell Viability Assay

To investigate the cytotoxicity of compounds, 2 × 10^4^ HEK293 cells were cultured in DMEM (+/+) on 96‐well flat‐bottom cell culture plates (Sarstedt, Germany) as described above. After 24 h, cells were treated with indicated concentrations of compounds (final DMSO concentration: 0.5%) and, if applicable, irradiated with a green LED lamp (*λ *= 520−530 nm; Eurolite, Germany) for 5 min. Control cells were treated with DMSO. Cell viability was measured 24 h after compound treatment. To this end, 25 µL Thiazolyl Blue Tetrazolium Bromide (MTT) solution (5 mg mL^−1^ MTT (Sigma Aldrich, USA) in PBS (Gibco, USA)) was added to each well directly into the medium. After 3 h of incubation at 37 °C, the medium was removed and formazan crystals were dissolved by adding 150 µL DMSO (1 h, 37 °C, gentle shaking), followed by an absorbance measurement at 570 and 630 nm as a reference using a Tecan Spark 10 M multimode microplate reader (Tecan Group Ltd., Switzerland). Each experiment was performed with five replicates. Relative cell viability was expressed as a percentage relative to the DMSO‐treated control cells.

## Conflict of Interest

The authors declare no conflict of interest.

## Supporting information

Supplementary Material

## Data Availability

Data available in article supplementary material.
