# Supplementary material for: A Photocaged N‐Phosphonopiperidinone as a Selective Photo‐Cleavable DPP8/9 Inhibitor
Source: Chembiochem. 2025 Sep 12;26(19):e202500558. doi: 10.1002/cbic.202500558 (PMC12498190; doi:10.1002/cbic.202500558)
Supplement: Supplementary file 1 — Supplementary Material [file CBIC-26-e202500558-s001.pdf]

Supplementary Information

A Photocaged *N*-Phosphonopiperidinone as a Selective Photo-Cleavable DPP8/9 Inhibitor

Leonard Sewald[+], Niko Molke[+], Werner W. A. Tabak, Anette Haak, Maja Najdzion, Ruth Geiss-Friedlander, Doris Hellerschmied, Robert Huber, Markus Kaiser*

*Corresponding author

[+]These authors contributed equally

Email: markus.kaiser@uni-due.de

Contents

Supplementary Schemes 3

Supplementary Figures 4

Supplementary Tables 5

Supplementary Methods 6

Chemical Synthesis 6

General Information 6

Synthetic Procedures 8

NMR Spectra 12

Supplementary References 16

Supplementary Schemes

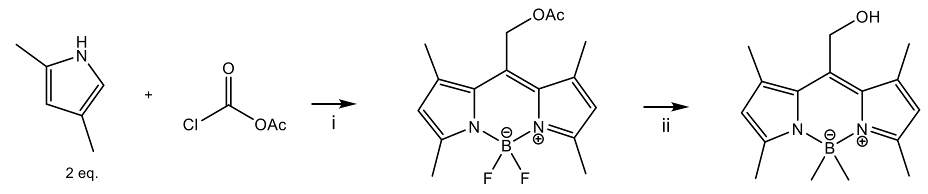

SI-1 **3**

Supplementary Scheme 1. Synthesis of the BODIPY photocage building block **3**.

(i) 1) DCM, 24 h, rt 2) DIEA, $BF_3 \cdot Et_2O$, 24 h, 0 °C; (ii) DCM, etheric 3 M MeMgBr, 2h, 0 °C

Supplementary Figures

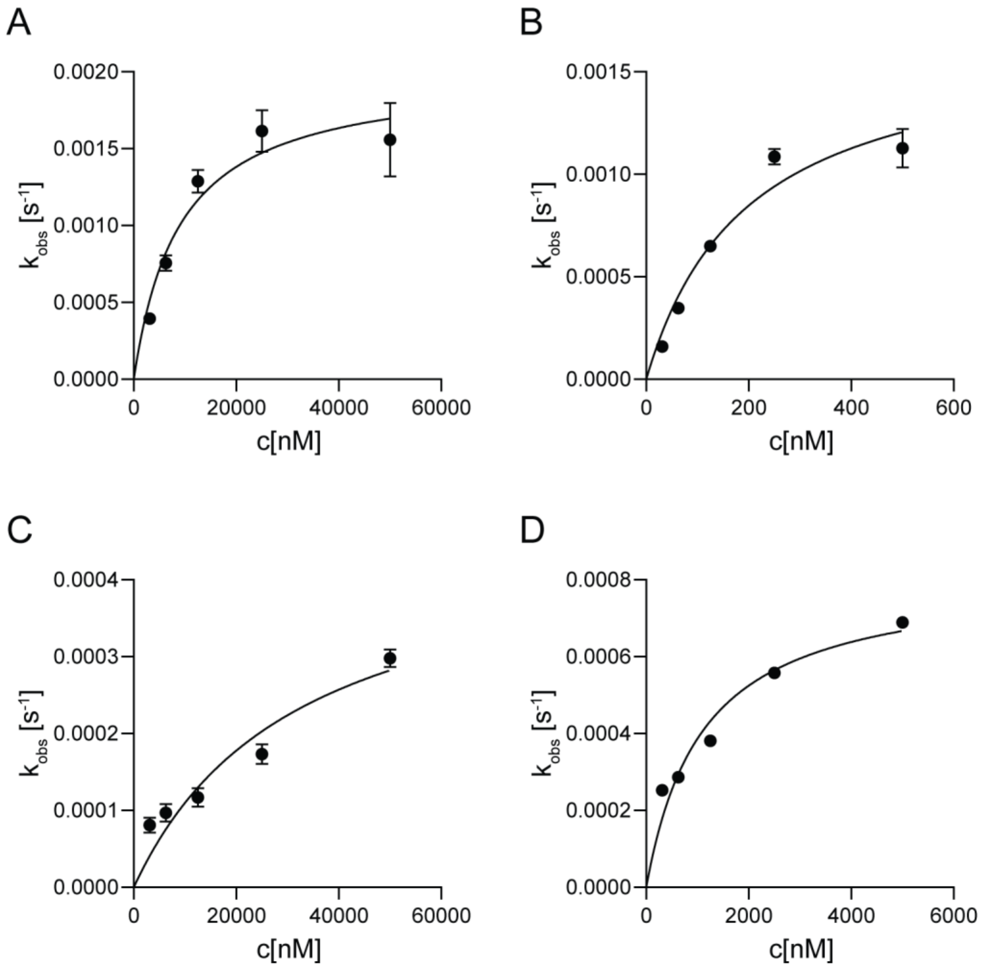

Supplementary Figure 1. Kinetic analysis of DPP8 and DPP9 inhibition by compound **5**. The pseudo-first order rate constant (k_{obs}) was calculated from an exponential regression of progress curves and plotted against the concentration of compound **5**. All measurement were performed in triplicate (*n* = 3 technical replicates), mean values are shown and error bars indicate the standard error of the mean (SEM). Inhibition assay was performed with DPP8 (A, B) and DPP9 (C, D) under 'dark' (A, C) and 'light' (B, D) conditions.

Supplementary Tables

Supplementary Table 1. Rate constants for irreversible inhibition of DPPs by **5** with or without irradiation.

		K_I [nM]	k_{inact} [s^{-1}]	k_{inact}/K_I [M^{-1} s^{-1}]	Dark/light ratio
DPP8	Dark	8,944 ± 2,037	0,001998 ± 0,000153	223	38
	Light	197 ± 42	0,001679 ± 0,000158	8,540	
DPP9	Dark	32,242 ± 10,332	0,000464 ± 0,000078	14	51
	Light	1,105 ± 171	0,000814 ± 0,000046	737	

Supplementary Methods

Chemical Synthesis

General Information

Chemicals and Other Materials.

All reagents were purchased from ABCR, Acros Organics, BLDPharm, Carbolution Chemicals, Carl Roth, Fisher, Merck, Sigma Aldrich, TCI Chemicals or VWR Chemicals and were used without further purification. All dry solvents were purchased from Acros Organics.

Column chromatography.

Compound purification by column chromatography was achieved using glass columns filled with silica gel (particle size 35 – 70 µm, from Acros Organics) as stationary phase and eluent mixtures of different solvents as mobile phase. The exact ratios of the solvents are listed in the corresponding synthesis procedures.

Thin layer chromatography (TLC).

Thin layer chromatography was performed on silica coated aluminum plates (60 F_{254}) from Merck. Detection of substances was conducted with UV light (wavelength 254 nm or 366 nm). The resulting R_f values including the used solvents are listed in the corresponding synthesis procedures.

HPLC and Mass Spectrometry.

Prep HPLC

Compound purification by HPLC was achieved using the Prominence UFLC system from Shimadzu (peak detection at 210 nm and 254 nm). The system was equipped with a reversed-phase C18 column from Phenomenex (Luna® 5 µm C18(2), 100 x 21.20 mm). For purification a linear gradient of solvent B (0.1 % TFA in acetonitrile) in solvent A (0.1 % TFA in water) at a flow rate of 15 mL min^{-1} was used.

LC-MS

LC-MS analysis were carried out with the setup described before, except analytes were separated using a 12 min gradient of solvent A (0.1% formic acid (FA) in MS-grade water) and solvent B (0.1% FA in ACN) (start with 10% B for 0.5 min, gradient 10% to 100% B for 5.5 min, 100% B for 3.2 min, gradient 100% to 10% B for 0.4 min, then 10% B) with a flow rate of 1 mL min^{-1}.

Nuclear Magnetic Resonance.

Nuclear magnetic resonance (NMR) spectra were recorded on a *Bruker Avance II 400* (400 MHz for ^1H NMR and 100 MHz for ^{13}C NMR) or *Bruker AV NEO 400 MHz* (400 MHz for ^1H and 100 MHz for ^{13}C NMR) machine. As solvents deuterated chloroform-d1 or deuterated DMSO-d6 were used. The chemical shifts δ are reported in parts per million (ppm). The spectra were referenced to the residual signals of undeuterated solvents (CDCl3: δ (^1H) = 7.26 and δ (^{13}C) = 77.16, DMSO: δ (^1H) = 2.50 and δ (13C) = 39.52, MeOD: δ (^1H) = 4.87 and δ (^{13}C) = 49.00). The coupling constants J are reported in Hertz (Hz). The ^1H NMR spectral data list the chemical shifts δ, the multiplicities (s: singlet, d doublet, t: triplet, q: quartet p: pentet, m: multiplet), the coupling constant J and the number of protons. The ^{13}C NMR spectra list only the chemical shifts δ and, if applicable, doublet multiplicity.

Synthetic Procedures

Synthesis of **SI-1**

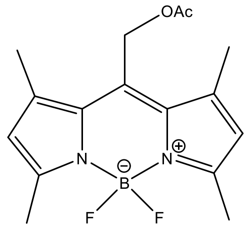

To a stirred solution of 2,4-dimethylpyrrole (4990 mg, 52.5 mmol, 2.0 eq.) in 450 mL dry DCM was added acetoxyacetyl chloride (4475 mg, 32.8 mmol, 1.3 eq.). After stirring overnight, triethyl amine (16.9 mL, 170.5 mmol, 7.5 eq.) was added at 0 °C and stirred for 20 min, followed by slow addition of boron tribromide etherate (29.8 mL, 209.8 mmol, 8 eq.). The following day all volatile components were removed under reduced pressure and the crude product purified by column chromatography. The product was obtained as a dark red solid (2018 mg, 24 %).

TLC (cyclohexane/DCM 1:2) R_f=0.56; **^{1}H-NMR** (400 MHz, [d6]DMSO): ***δ***=6.08 (s, 2H), 5.29 (s, 2H), 2.53 (s, 6H), 2.36 (s, 6H), 2.13 ppm (s, 3H); **^{13}C-NMR** (101 MHz, [d6]DMSO) ***δ***=170.55, 156.66, 141.47, 133.34, 132.69, 122.33, 57.87, 20.58, 15.61, 14.68, 14.66 ppm; **LC-MS (ESI)** *m/z* calcd for $C_{16}H_{19}BF_2N_2O^+$: 320.15 [*M*]$^+$; found 320.15; t_R = 8.41 min. The analytic data were in accordance with prior published data.[1-2]

Synthesis of **3**

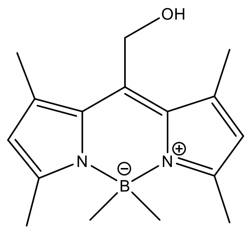

To a solution of **SI-1** (112.9 mg, 0.35 mmol, 1 eq.) in 60 mL dry DCM was slowly added etheric MeMgBr (3 M, 1.76 mL, 5.3 mmol, 15 eq.). After the reaction was completed, saturated ammonium chloride solution was carefully added to quench unreacted MeMgBr. The reaction mixture was diluted with ethyl acetate and the organic phase washed with saturated ammonium chloride three times and dried over sodium sulfate. The crude product purified by column chromatography. The product was obtained as a red powder (34 mg, 35 %).

TLC (DCM) R_f=0.13 ; **^{1}H-NMR** (400 MHz, [d6]DMSO): ***δ***=6.09 (s, 2H, 4.96 (s, 2H), 2.53 (s, 6H), 2.46 (s, 6H), 0.18 (s, 6H) ppm (s, 3H); **LC-MS (ESI)** *m/z* calcd for $C_{16}H_{32}BN_2O^+$: 271.20 [*M*+H]$^+$; found 271.19; t_R = 8.41 min. The analytic data were in accordance with prior published data.[1-2]

Synthesis of **4**

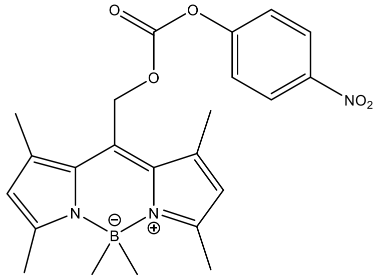

Compound **3** (158.2 mg, 0.59 mmol, 1 eq.) and DIEA (1019 µL, 5.9 mmol, 10 eq.) were dissolved in 15 mL dry DCM. In a second flask, 4-nitrophenyl chloroformiate (1177 mg, 5.9 mmol, 10 eq.) was dissolved in 10 mL dry DCM. After addition of pyridine (473 µL, 5.9 mmol, 10 eq.) a white precipitate formed. Both flasks were cooled to 0 °C and the white suspension was added to the solution of compound **3**. After stirring for 16 h at room temperature, the reaction mixture was diluted with DCM, washed three times with 0.1 N HCl, two times water and once with brine and dried over sodium sulfate. Compound **4** was purified by column chromatography (cyclohexane/DCM). The product was obtained as a red solid (136 mg, 53 %).

TLC (cyclohexane/DCM 1:1*)* R_f=0.26; **^{1}H-NMR** (400 MHz, [d6]DMSO): ***δ***=8.29 (d, J = 9.1 Hz, 2H), 7.40 (d, J = 9.1 Hz, 2H), 6.12 (s, 2H), 5.61 (s, 2H), 2.48 (s, 6H), 2.46 (s, 6H), 0.20 (s, 6H); **^{13}C-NMR** (101 MHz, [d6]DMSO) ***δ***=155.49, 153.96, 152.44, 145.64, 137.06, 131.01, 125.52, 123.28, 121.75, 62.77, 16.78, 16.21, 9.64 ppm; **LC-MS (ESI)** *m/z* calcd for $C_{23}H_{26}BN_3O_5^+$: 436.20 [*M*+H]$^+$; found 436.25; t_R = 9.76 min. The analytic data were in accordance with prior published data.[1-2]

Synthesis of **5**

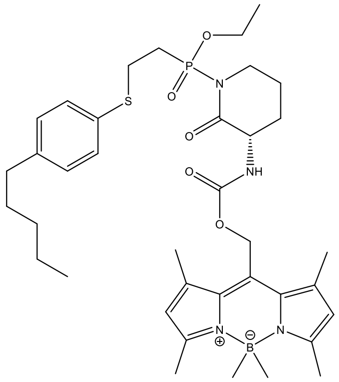

To a stirred solution of **4** (6.33 mg, 14.6 µmol, 1.5 eq.) in 100 µL dry DMSO was added compound **1** (prepared as described in Sewald *et al.*;[3] 6.00 mg, 9.4 µmol, 1.55 eq.) and DIEA (5 µL, 29 µmol, 3.1 eq.). After the reaction was finished, the reaction was diluted with water and extracted with diethyl ether, until the aqueous phase was almost colorless. The organic phase was dried over sodium sulfate, dark residue purified via HPLC. After lyophilization, the product was obtained as a red-orange powder (4.56 mg, 69 %)

^{1}H-NMR (400 MHz, [d6]DMSO): ***δ***=7.74 (dd, J = 10.6, 8.4 Hz, 1H, *H*38), 7.25 (dd, J = 8.2, 2.6 Hz, 2H, Ar*H*), 7.15 (dd, *J* = 7.8, 7.8 Hz, 2H, Ar*H*), 6.20 (s, 2H, *H*8 and *H*11), 5.25 (d, J = 11.9 Hz, 2H, *H*13), 4.16 (dt, J = 18.6, 7.6 Hz, 1H, *H*36), 4.01 – 3.93 (m, 1H, *H*30'), 3.92 – 3.78 (m, 1H, *H*30''), 3.58 (t, J = 5.7 Hz, 2H, *H*33), 3.04 (tt, J = 10.7, 7.5 Hz, 2H, *H*25), 2.57 – 2.50 (m, 2H, *H*45), 2.41 (s, 6H, *H*14-17), 2.37 (s, 3H, *H*14-17), 2.36 (s, 3H, *H*14-17), 2.26 – 2.16 (m, 2H, *H*26), 2.04 (td, J = 13.0, 6.7 Hz, 1H, *H*37''), 1.83 – 1.79 (m, 2H, *H*32), 1.71 – 1.66 (m, 1H, *H*37'), 1.53 (dp, J = 15.9, 7.8 Hz, 2H, *H*46), 1.32 – 1.28 (m, 2H, *H*48), 1.30 – 1.21 (m, 2H, *H*47), 1.18 (td, J = 7.0, 2.8 Hz, 3H, *H*31), 0.84 (td, J = 7.0, 3.2 Hz, 3H, *H*49), 0.11 ppm (s, 6H, *H*18/22);**^{13}C-NMR** (101 MHz, [d6]DMSO) ***δ***=173.52 (*C*35), 155.80, 152.46, 140.75 (*C42*), 137.40 (*C*7/12), 134.51 (C_q-BODIPY), 131.53 (*C*23). 130.64 (C_q-BODIPY), 129.27 (*C*40/44), 129.25 (*C*40/44), 129.17 (*C*41/43), 122.64 (*C*8/11), 60.62 (*C*30), 57.90 (*C*13), 51.57 (*C*36), 43.09 (d, *J* = 27.3 Hz, *C*33), 34.60 (*C*45), 30.87 (C47), 30.53 (*C*46), 26.98 (*C*26), 25.65 (*C*25), 21.94 (*C*48), 21.12 (*C*32) 20.94 (d,*C*37), 16.21 (*C*14-17), 16.05 (*C*14-17), 15.98 (*C*31), 15.47 (*C*14-17), 13.91 (*C*49), 10.09 (*C*18/22); **^{31}P-NMR** (162 MHz, [d6]DMSO) = 28.55, 28.50 ppm; **LC-MS (ESI)** *m/z* calcd for $C_{37}H_{54}BN_4O_5PS^+$: 709.37 [*M*+H]$^+$; found 709.43; t_R = 11.59 min; **HRMS (ESI):** *m/z* calcd. for $C_{37}H_{54}BN_4O_5PS^+$: 709.3724 [*M*+H]$^+$; found 709.3698 (|Δ| = 3.67 ppm) 413.2008 [M-BODIPY]

NMR Spectra

Compound **5**

^{1}H-NMR

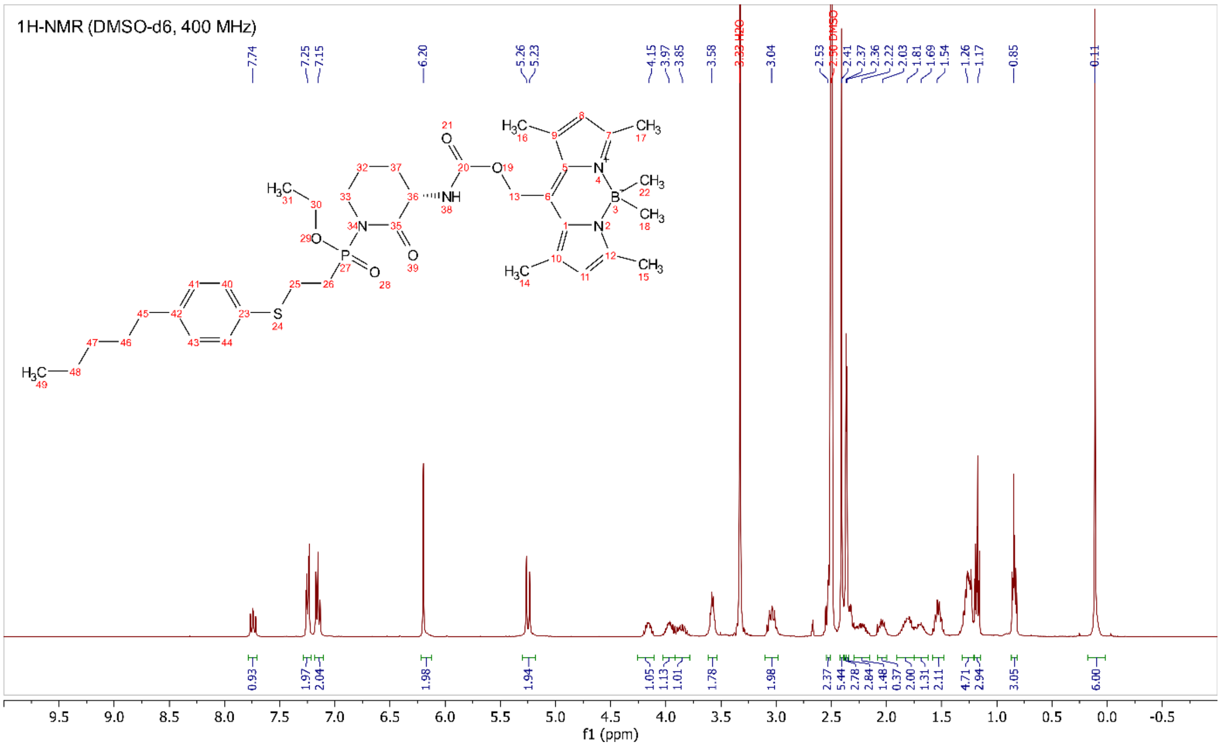

^{13}C-NMR

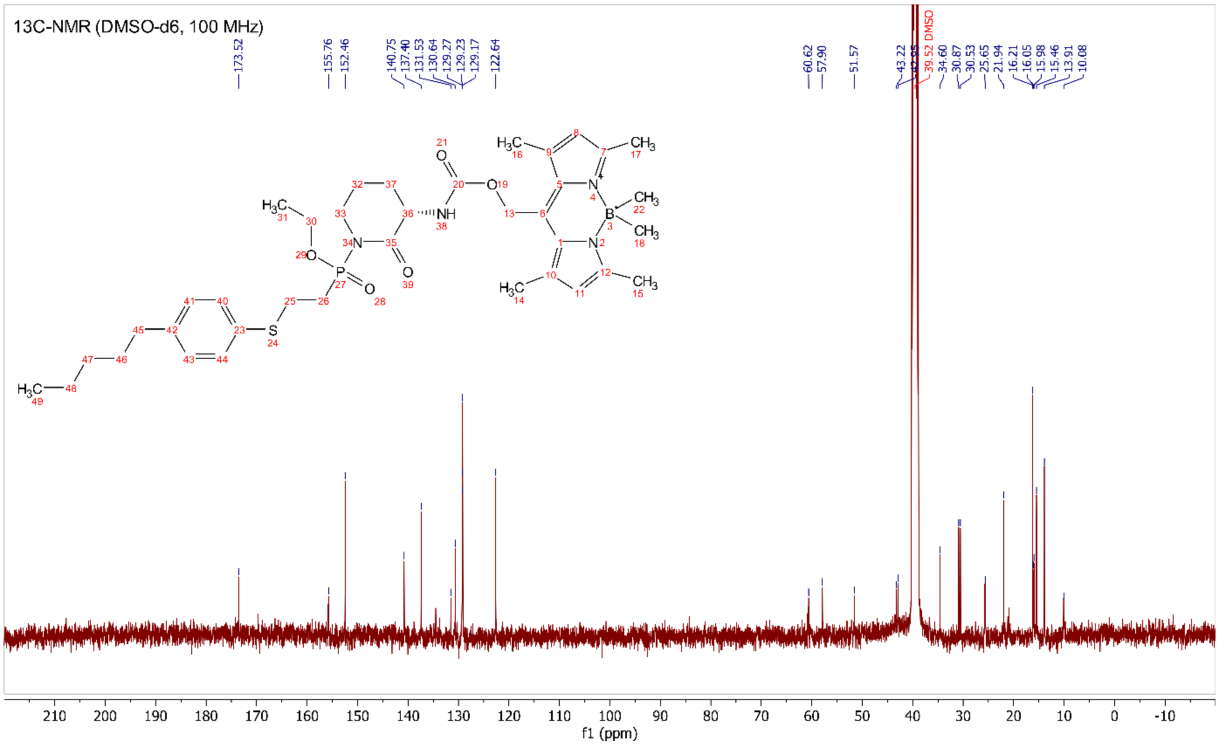

^{31}P-NMR

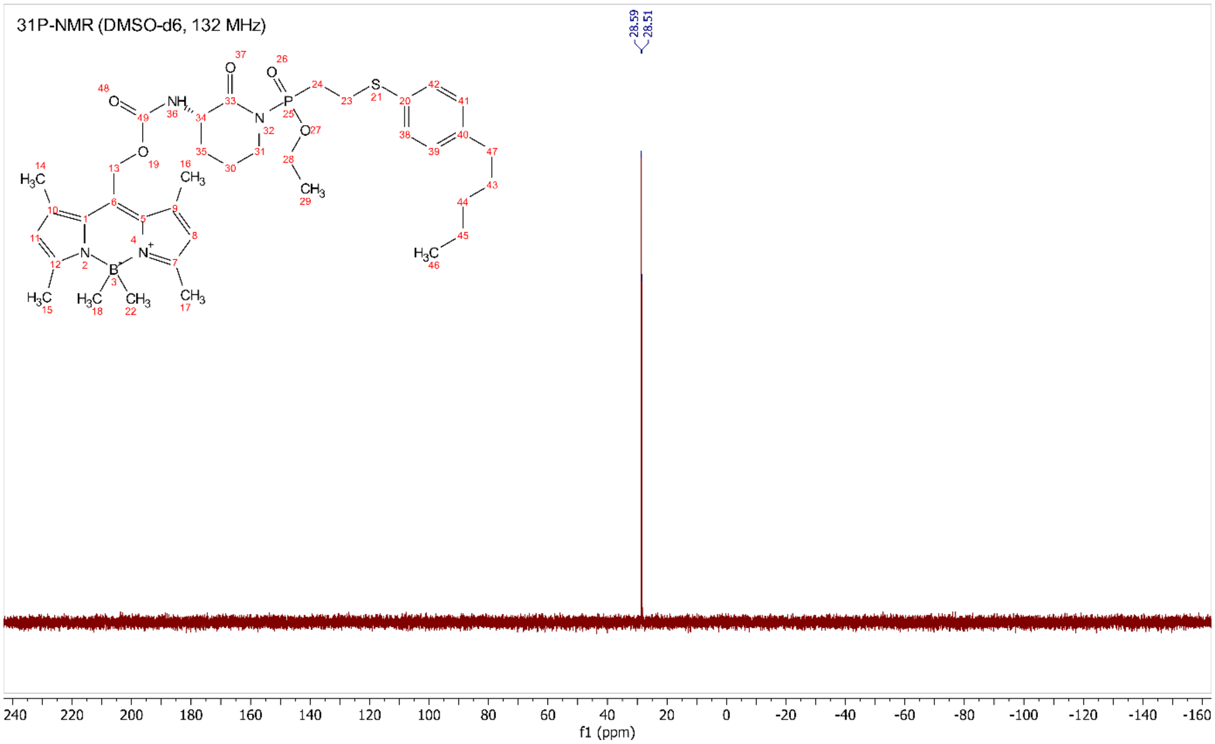

$^{1}H,^{1}H$-COSY

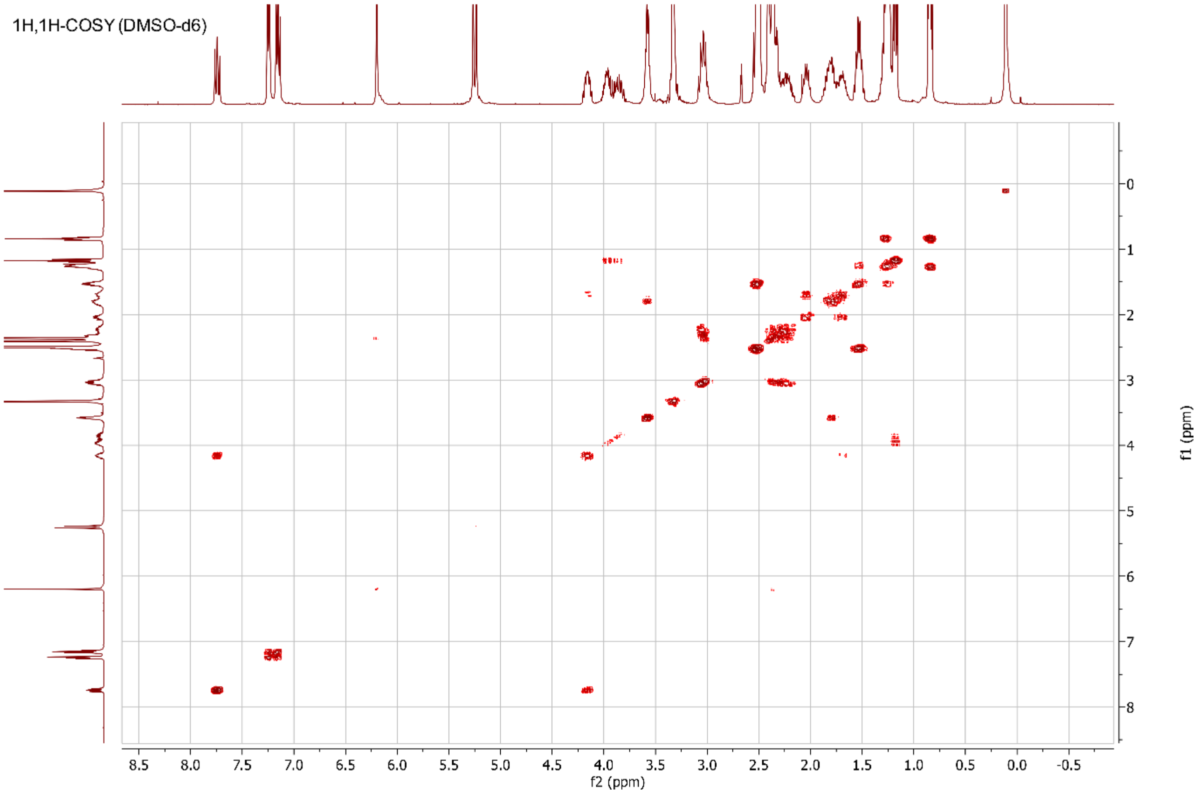
1H,1H-COSY (DMSO-d6)
8.5
8.0
7.5
7.0
6.5
6.0
5.5
5.0
4.5
4.0
3.5
3.0
2.5
2.0
1.5
1.0
0.5
0.0
-0.5
f2 (ppm)
0
1
2
3
4
5
6
7
8
f1 (ppm)

HMBC

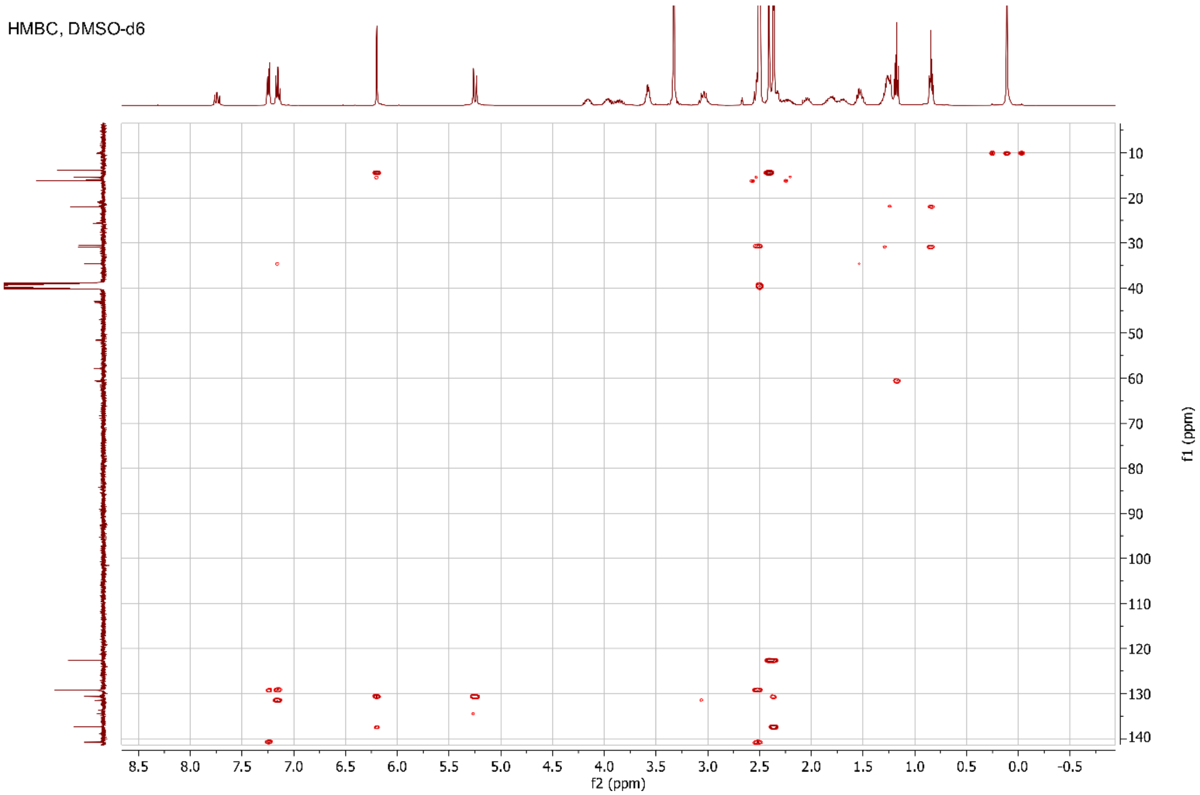

$^{1}H,^{13}C$-HSQC

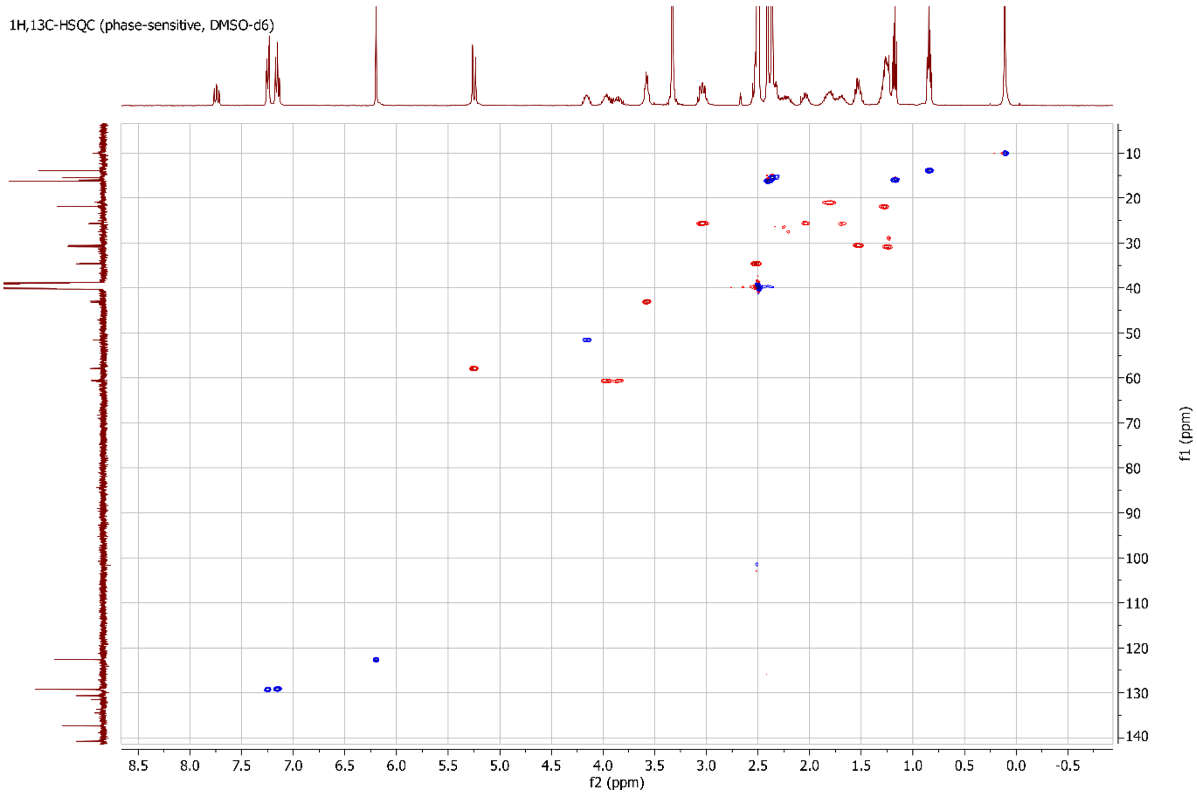

Supplementary References

[1] T. Slanina, P. Shrestha, E. Palao, D. Kand, J. A. Peterson, A. S. Dutton, N. Rubinstein, R. Weinstain, A. H. Winter, P. Klán, *J Am Chem Soc* **2017**, *139*, 15168-15175.

[2] P. Shrestha, A. Mukhopadhyay, K. C. Dissanayake, A. H. Winter, *J Org Chem* **2022**, *87*, 14334-14341.

[3] L. Sewald, W. W. A. Tabak, L. Fehr, S. Zolg, M. Najdzion, C. J. A. Verhoef, D. Podlesainski, R. Geiss-Friedlander, A. Lammens, F. Kaschani, D. Hellerschmied, R. Huber, M. Kaiser, *Nat Commun* **2025**, *16*, 3208.
